# Development of electronic medical record charting for hospital-based transfusion and apheresis medicine services: Early adoption perspectives

**DOI:** 10.4103/2153-3539.65345

**Published:** 2010-07-13

**Authors:** Rebecca Levy, Liron Pantanowitz, Darlene Cloutier, Jean Provencher, Joan McGirr, Jennifer Stebbins, Suzanne Cronin, Josh Wherry, Joseph Fenton, Eileen Donelan, Vandita Johari, Chester Andrzejewski

**Affiliations:** 1 Department of Pathology, Tufts University School of Medicine, Baystate Medical Center, Springfield, MA, USA; 2 Department of Nursing, Tufts University School of Medicine, Baystate Medical Center, Springfield, MA, USA; 3 Department of Information Services, Tufts University School of Medicine, Baystate Medical Center, Springfield, MA, USA

**Keywords:** Apheresis, blood bank, electronic medical record, informatics, transfusion

## Abstract

**Background:**

Electronic medical records (EMRs) provide universal access to health care information across multidisciplinary lines. In pathology departments, transfusion and apheresis medicine services (TAMS) involved in direct patient care activities produce data and documentation that typically do not enter the EMR. Taking advantage of our institution's initiative for implementation of a paperless medical record, our TAMS division set out to develop an electronic charting (e-charting) strategy within the EMR.

**Methods:**

A focus group of our hospital's transfusion committee consisting of transfusion medicine specialists, pathologists, residents, nurses, hemapheresis specialists, and information technologists was constituted and charged with the project. The group met periodically to implement e-charting TAMS workflow and produced electronic documents within the EMR (Cerner Millenium) for various service line functions.

**Results:**

The interdisciplinary working group developed and implemented electronic versions of various paper-based clinical documentation used by these services. All electronic notes collectively gather and reside within a unique *Transfusion Medicine Folder* tab in the EMR, available to staff with access to patient charts. E-charting eliminated illegible handwritten notes, resulted in more consistent clinical documentation among staff, and provided greater realered. However, minor updates and corrections to documents as well as select work re-designs were required for optimal use of e-charting-time review/access of hemotherapy practices. No major impediments to workflow or inefficiencies have been encount by these services.

**Conclusion:**

Documentation of pathology subspecialty activities such as TAMS can be successfully incorporated into the EMR. E-charting by staff enhances communication and helps promote standardized documentation of patient care within and across service lines. Well-constructed electronic documents in the EMR may also enhance data mining, quality improvement, and biovigilance monitoring activities.

## INTRODUCTION

The electronic medical record (EMR) has existed for well over a decade and is expanding increasingly in the health care setting. The EMR provides a repository of patient encounters, problem lists, clinical notes, procedures, test results, and helps alleviate tedious and labor intensive filing and retrieval inefficiencies associated with paper-based records. When well-designed and implemented, the benefits of an EMR can outweigh the disadvantages.[1,2] Recognized benefits include enhancements in patient safety, error reduction (e.g. adverse drug events), promotion of standardized (protocol-based) patient care, portability of information and handling of mundane administrative functions (e.g. automated coding).[3–5] In contrast, paper records are cumbersome to store/convey, are not readily transportable, may contain illegible entries, lack select clinical service line documents or may be simply unavailable because of simultaneous provider use. Lost or misplaced documents also characterize paper-based systems and according to one study, paper charts may be missing up to 25% of the time when they are clinically needed.[6]

Within the EMR, computerized data input also improves data quality and validity. For example, handwritten records can be associated with poor legibility that may contribute to medical errors. Structured data entry and electronic templates prompt clinicians to provide complete information. Electronically stored laboratory data in the EMR can be more easily accessed and managed, and offer potential advantages in new designs of innovative clinical patient monitoring and quality improvement reporting. Users can rapidly seek, view, arrange and assemble laboratory information to support trend analyses and clinical decision making.

Clinical decision support functions within EMR systems allow for more timely, robust and interactive contributions in multidisciplinary clinical care decision making processes. Such tools may include clinical alerts (e.g. pop-up notices), reminders, formula calculations and protocol–driven order sets. With integration of institutional laboratory information system (LIS) operations into the EMR, the acceptance of laboratory test orders via computerized physician order entry (CPOE) systems and/or electronic reporting of test results to the EMR are all more readily achieved.[7] Educational content may also be embedded within the EMR.

Given these many facets of an EMR, the adoption of an electronic health record by hospitals could enhance value added contributions from pathology laboratories in clinical decision making processes. To the best of our knowledge, however, there have been limited applications of the EMR in the practice of transfusion medicine.[8] Unlike other areas of pathology, the transfusion and apheresis medicine service (TAMS) not only performs diagnostic activities, but also actively participates therapeutically in patient care activities either directly or indirectly via the transfusion process or the performance of hemapheresis procedures, blood collections and therapeutic phlebotomies. Such activities, typically with direct patient care encounters, involve consultations, obtaining patient consent and histories as well as the performance of physical examinations and therapeutic procedures. Integration of these activities into a hospital EMR, although challenging, could allow not only the TAMS division to benefit from many of the aforementioned advantages of an integrated EMR, but also clinical colleagues as well.[9,10]

Historically, at our institution clinical records for TAMS activities consisted primarily of paper-based documentation. For example, patient monitoring during a blood transfusion was recorded by nursing staff solely on paper charts. Suspected transfusion reaction investigation reports would need to be typed, printed on paper and delivered to pertinent parties. Since these text-based (qualitative) reports could not be created in our blood bank LIS (HCLL, Mediware Information Systems, Inc., Oak Brook, IL, USA), unless they were electronically scanned, they were not being incorporated into the patient's chart in the EMR. At some institutions, such reports (e.g. synoptic apheresis reports) have been incorporated into the Anatomical Pathology LIS as a mechanism of transmitting them to the EMR in a timely manner.[11] Difficulties in document storage and retrieval for our clinically busy TAMS were commonly encountered, especially due to limitations in the physical spaces allocated to the operations of these service lines.

In light of the advantages afforded by an EMR, along with a hospital systemwide directive promoting the adoption of electronic records, we undertook an initiative to incorporate TAMS activities directly into our hospital's EMR. Herein, we describe our early efforts in this regard and lessons learned in the experience.

## METHODS

### Clinical Setting

Baystate Health (BH) is an integrated health care delivery system located in Springfield, MA, USA, with a patient care network covering the western third of the state of Massachusetts. BH consists of three hospitals, multiple clinics and physician practices. Baystate Medical Center (BMC), the largest hospital in the system and the third largest in the Commonwealth of Massachusetts, is a 650-bed academic/community hospital that serves as the western campus of the Tufts University School of Medicine offering clinical teaching of medical students, residents and fellows. BMC is a level 1 trauma center with an integrated multispecialty cancer center and a renal transplant program. Cerner Millennium (version 2007.19, Cerner Corporation, Kansas City, MO, USA) is the EMR platform for our regional health system and is accessed via networked workstations throughout our care network. This EMR system interfaces with various LISs including the Clinical Pathology LIS (Sunquest, Sunquest Information Systems, Tucson, AZ, USA), the Anatomical Pathology LIS (CoPath Plus, Cerner Corporation, Kansas City, MO, USA), and the blood bank LIS (HCLL Transfusion, Mediware Information Systems, Inc.). The transfusion medicine service (TMS) annually crossmatches over 26,000 units of blood, issues around 24,000 units of various blood products for transfusions, and evaluates approximately 300 suspected transfusion reactions. The apheresis medicine service (AMS) performs over 500 hemapheresis procedures annually. The blood bank has been computerized since 1985. Electronic cross-match was adopted in August 1994.

### Focus Group Activities

As part of an ongoing health care operation's quality improvement initiative of the TMS, focusing on bedside biovigilance as it relates to the recognition and reporting of suspected transfusion reactions (STRs), re-design of the paper-based blood product transfusion identification/documentation tag was started in January 2007 by a focus group of our hospital's transfusion committee. Members of the Clinical Informatics and Information Services (IS) department were engaged to simultaneously develop an electronic chart version of the tag as part of a hospital-wide directive regarding the adoption of an EMR. The scope of the project was expanded to include the development of clinical e-documentation for both TMS and AMS. The working group was composed of pathologists (both attending and resident staff), nursing staff, hemapheresis specialists, laboratory technologists, various clinical informaticists including an application engineer, application analyst and a clinical informatics specialist. The group met regularly to discuss the clinical “care maps” (procedure plan) for hemotherapies[12] and hemapheresis, logistics of inpatient and outpatient transfusions, system software and hardware requirements, budget-related issues, report format designs, potential impact on staff and workflow, and the display options for the created documents including the construction of a unique TMS folder (electronic tab) in our EMR. Attention was also given to future data mining options and opportunities, especially as they related to blood use review and apheresis activities.

The group met periodically to vet various iterations of electronic forms. Although many of the e-documents were available within 12 months of project initiation, the decision for a phased implementation strategy was adopted rather than a “bolus approach”, or in IS terminology a “big bang”. All new software builds were piloted, continuously reviewed, revised, and workflow issues addressed and improved in the IS test environment before introduction into the EMR. Per focus group consensus, select service piloting of the documents in the actual (live) EMR environment was not included in the release strategy. Prior to “go-live” implementation dates, training of pertinent nursing and medical staff was conducted by IS personnel. Two to three months of staff training was targeted. Post go-live implementation, auditing/validation of documents was performed. This consisted of detailed reviews of randomly selected e-documents to ensure that they contained the appropriate and accurate charting information. In addition, IS staff monitored all electronic blood tag use through a Cerner CCL® report in the first week of that document's introduction. In situations where immediate corrective action was identified to correct aspects of the document, team members communicated directly with each other to remedy the situation. The focus group continued to meet periodically to discuss the need for upgrades and enhancements.

## RESULTS

### Development and Implementation Milestones

Within 12–16 months of project initiation, draft e-versions for an *Apheresis Medicine Consult Note, Apheresis Medicine Procedure Note, Apheresis Medicine Progress Note, Autologous Post-Operative Blood Recovery and Infusion Note* and *Blood Product Transfusion Tag* were designed. Factors that inhibited immediate implementation included lack of mobile computer hardware for the apheresis service, allocation of training time for busy staff, limitation of the EMR for select functions (e.g. double witness attestations for the e-blood tag) and these had to be resolved before e-charting could be embraced. Moreover, there were numerous competing projects and EMR version upgrades. Once these competing demands were resolved, e-documentation implementation proceeded stepwise with release of the e-documents in the following order: *Apheresis Medicine and Transfusion Medicine Speciality Flowcharts* (06/2008); *Apheresis Medicine Consult Note* (08/2009); *Apheresis Medicine Procedure Note and Apheresis Medicine Progress Note* (11/2009); *Transfusion Folder Tab (12/2009); and Autologous Post-Operative Blood Recovery and Infusion Note and Blood Product Transfusion Tag (01/2010)*. Major attributes of some of the e-documents are described below.

### e-Charting Documentation

**Figure 1:**
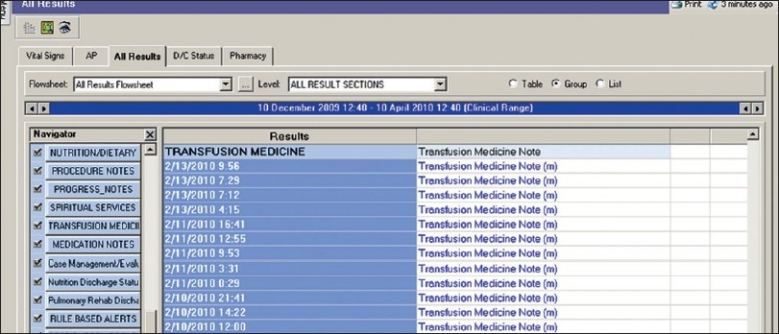
Display screen of select elements located within the Transfusion Medicine Folder tab of the Patient Care Documentation section of Clinical Information System (CIS)

*Transfusion Folder Tab*: This folder [Figure 1] is located within the Clinical Notes section of the EMR, under the Patient Care Documentation tab amongst the most commonly accessed documents in the EMR such as admission notes, operative notes and discharge/transfer summary notes. The TMS folder includes the *Apheresis Medicine Consult Note, Apheresis Medicine Procedure Note* and *Apheresis Medicine Procedure Record*. These electronic notes were designed to pull in selective past medical history (e.g. disease diagnosis, known allergies), medication lists, laboratory tests and vital sign values from other areas of the EMR into a truncated display format that could be included, if desired by the author of these notes. The *Blood Product Transfusion Tag Form* also appears is this section of the EMR.

**Figure 2:**
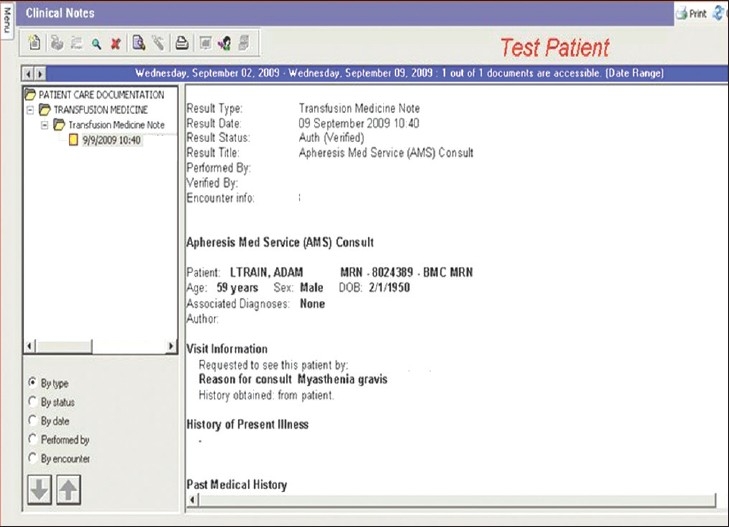
Display screen of select elements of the Apheresis Medicine Consult Note, located within the Transfusion Medicine folder of the Patient Care Documentation section of CIS

*Apheresis Medicine Consult Note*: The apheresis medicine physician consult note [Figure 2] is used by the AMS medical staff in their initial consultation when evaluations are made regarding the feasibility and indications for an apheresis intervention for a patient. It includes patient demographics, requesting/referring physician information, pertinent clinical history, allergies, selected medications, review of systems, physical examination results, pertinent laboratory values and an assessment/plan section.

**Figure 3:**
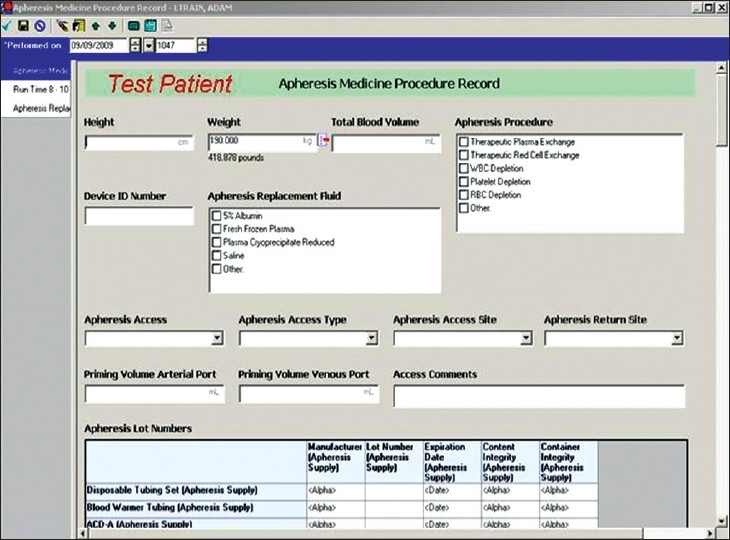
Display screen of select elements of the Apheresis Medicine Procedure Note, located within the Transfusion Medicine folder of the Patient Care Documentation section of CIS

*Apheresis Medicine Procedure Record*: This e-note [Figure 3] allows hemapheresis specialists and/or nurses to record data and information relevant to the apheresis procedure such as patient height, weight, total blood volume, targeted removal volume, type of apheresis procedure (e.g. therapeutic plasma exchange, red cell exchange, etc.), apheresis device and equipment used, replacement fluids given, patient vascular access details, instrument priming details, patient education documentation, vital sign values and start/end procedure run time. Data fields embedded in the document also permit one to record medications used during an apheresis as well as device, plasticware and replacement fluid identification/lot numbers including expiration dates. This permits the service to meet currently acceptable good manufacturing practices, allows for easier automated quality control monitoring, and enhanced biovigilance/patient safety in the event of product recall actions or potential “look back” investigations.

**Figure 4:**
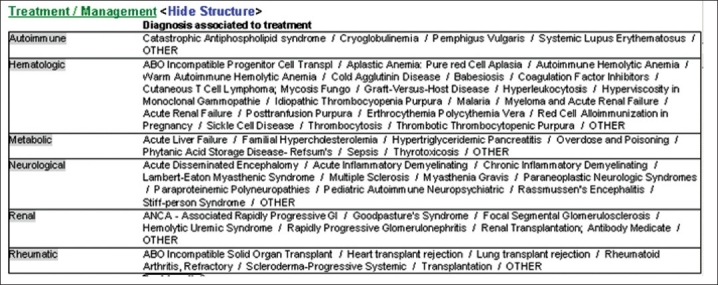
Display screen of diagnosis associated with treatment elements of the Apheresis Medicine Consult Note, located within the Transfusion Medicine folder of the Patient Care Documentation section of CIS

*AMS Therapeutic Plasma Exchange Note*: This multi-author e-document is used to record the clinical details associated with each separate apheresis medicine procedure. The note automatically keeps track of the number of apheresis procedures in a series of treatments. It incorporates a multitude of information including an ongoing patient specific problem list, procedurally related ordered medications, details regarding vascular access and catheter care, treatment and replacement fluid parameters, intra-procedural patient vital sign values, select pertinent laboratory values (nearest charted values), procedural complications, an assessment/plan/recommendations section and areas documenting health care providers involved in the intervention. This note is modifiable with respect to the different hemapheresis procedures performed. Depending on the hemapheresis procedure performed, each note has a different format and information included. The “Diagnosis Associated to Treatment” section of the note [Figure 4] listing the disease indication for the apheresis procedure is predicated on the currently recognized disorders amenable to treatment with hemapheresis interventions set forth by the American Society for Apheresis.[13]

**Figure 5:**
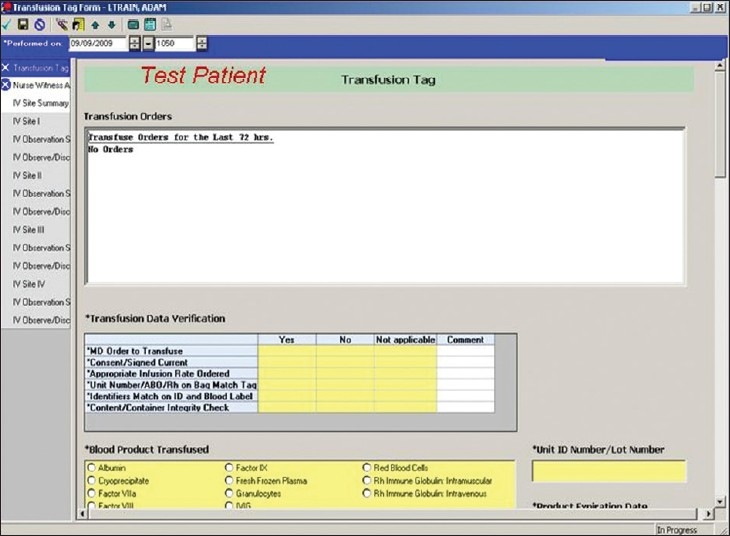
Display screen of select elements of the Blood Product Transfusion Tag, located within the Transfusion Medicine Folder of the Patient Care Documentation section of CIS

*Blood Product Transfusion Tag Form*: This electronic form [Figure 5] serves as the preferred documentation route for a blood transfusion event in clinical care areas with access to the EMR. Although a pre-existing transfusion documentation area was present in the EMR version at the start of the project, it was considered to be cumbersome and insufficient (e.g. initially only one set of vital sign values could be charted). The new e-document was constructed to contain data fields for checklist items required in bedside review prior to the start of the transfusion including acknowledgement of patient consent and education, specific product and compatibility results, patient vital sign values at the start, the 15 minute interval, and the end of the transfusion event, as well as the recording of a potential suspected transfusion reaction and its associated signs and symptoms. Recording a double witness attestation is integral to the form and was designed to meet stringent safety requirements. Although the double witness attestation should occur immediately prior to the transfusion, its documentation by the witness may not occur in the e-record until a later time depending on work-flow demands. This will not prevent the start of the transfusion; however, its documentation will remain as an open item on the nursing task list until it is completed. If such action is not accomplished within 24 hours of the transfusion, automatic notification regarding this undocumented task is forwarded to the Clinical Informatics division for follow-up and compliance monitoring. The development of this particular area on the form took a considerable amount of time and effort and was one reason for the form's delayed introduction.

### Post Implementation Documentation Revisions

After implementation, minor changes related to the display of data, corrections for units of measure and enhancements in the process of forwarding notes for electronic signature were needed. In addition, problems related to incorporation of surplus and unnecessary data via the auto-population process of laboratory test values and vital sign values within documents were subsequently corrected. This was accomplished by limiting the time interval to pull in additional data, making the notes more manageable. These finer details were only realized after individuals began routine documentation within the EMR. The inability of the EMR to support a medical word spell-check function was identified as another process that needs to be addressed in the future.

## DISCUSSION

Over the past 3 years our TAMS division has successfully developed and implemented electronic charting in the EMR of documentation not previously available in our LIS. This accomplishment resulted from the sustained efforts of an interdisciplinary working group of individuals with talents and expertise pertinent to the targeted charges of developing e-charting in select pathology subspecialty areas. Project delays in implementation were related to a variety of factors including challenges related to software development, necessary hardware acquisitions and competing project demands. Other institutions considering the development of an EMR that includes aspects of their TAMS may find many of the same types of challenges we encountered in this project.

Our electronic design of the *Blood Product Transfusion Tag* allows all pertinent patient and infusion information regarding a specific transfusion event to be readily available in the EMR. This includes the volume of intravenous fluids a patient receives prior to and during a transfusion, vital sign value changes during infusion, and adverse signs or symptoms, if any, that the patient exhibits during hemotherapy. In the event of a suspected transfusion reaction, electronically captured data are communicated automatically to the TMS division. This, in turn, triggers an order for a suspected transfusion reaction investigation. This greatly facilitates the work-up in the blood bank, thereby saving time, reducing repetitive clerical work and promoting standardization in such patient diagnostic work-ups.

The advantage of having all of the aforementioned information available in the EMR not only benefits the clinical teams treating these patients, but also has proved to be immensely helpful for the pathology residents and pathologists involved in the work-up of these cases. Pathology residents and attendings have been able to securely and simultaneously access TAMS documents from different locations, including their home computers. Having pertinent patient transfusion history, prior transfusion/apheresis procedures and current management plans universally available online has been particularly helpful for pathologists participating in after duty hours/weekend on-call coverage of these service lines. Additionally, using the EMR in this manner helps solve several problems with paper charting: lost or misplaced charts, illegible writing and the existence of only one official copy of a handwritten chart note. The creation of a transfusion medicine folder that houses all these data within the EMR in one location is, in our opinion, therefore an effective manner in which to timely convey the information within our own TAMS division as well as to clinicians and contribute to enhance interdisciplinary communications that are critical to improving patient care.

Although we are yet to perform definitive time studies on the use of these various forms and the workloads associated with them, through informal conversations with nurses as well as our own clinical e-charting experience, some increase in task accomplishment times has been identified by staff. Whether this represents a real increase in time spent by individuals doing these activities or this is just part of the learning curve associated with new skills development remains to be determined. Interestingly, leadership of the AMS believes that some efficiencies with regard to less duplication of paperwork has been achieved and therefore has resulted in less preparation time for the apheresis procedures.

The success of this project was largely dependent upon the integral roles of both specialty content experts and dedicated assigned staff from the Clinical Informatics and IS division, not only during the early developmental stages but also in the implementation phases of the project when “trouble-shooting” of the e-documents was needed. The importance of an integrated team approach involving content experts and technical experts skilled in informatics has been identified previously and our experience here confirms the wisdom of such a team endeavor[8,10] Both specialty areas of transfusion medicine and information technology are composed of a cadre of individuals with differing roles, expertise and responsibilities within the care-map of hemotherapy.[8,12] The inclusion of content experts from these various professional disciplines positively leverages problem-solving abilities of the team, offering unique insight, creative solutions and the necessary skills when problems/issues are encountered.[10]

We view electronic charting by our staff in the EMR as an evolving approach that helps to promote standardization of patient care and improve the biosafety of hemotherapies. In anticipation of the need for greater real-time monitoring of hemotherapy events, the discrete data elements included within our electronic notes have been selected and formatted to facilitate data mining of this information for quality improvement, blood utilization review and biovigilance regulatory purposes. With TAMS data now in the EMR, these services can begin to consider developing helpful decision support tools (e.g. alert systems).[14] Indeed, we are looking at creating such decision support tools within the EMR to generate electronic alerts prior to a transfusion for those individuals within our health care system who may be at increased risk of transfusion associated fluid overload (TACO),[15,16] or who have had prior transfusion reactions. We believe that the work described here adds to the existing but limited pathology literature detailing approaches that pathology departments must make to be active participants in the development of clinical electronic records that incorporate critical care elements derived from pathology service lines, especially those in the clinical pathology areas.

In summary, over a 3-year time period, we were able to successfully incorporate TAMS activities at our institution into our EMR. This has promoted documentation standardization within these care areas. As a result, the EMR now serves as a repository for TMS and AMS activities at our institution and allows individuals at many different locations to have immediate access to this information. Future studies are needed to determine how and to what extent these e-charting efforts will improve patient safety associated with service lines supporting transfusion and apheresis medicine activities.
